# Effectiveness of an mHealth- and School-Based Health Education Program for Salt Reduction (EduSaltS) in China: Cluster Randomized Controlled Trial Within Scale-Up

**DOI:** 10.2196/60092

**Published:** 2025-03-27

**Authors:** Naibo Wang, Chen Wang, Puhong Zhang, Yinghua Li, Feng J He, Li Li, Yuan Li, Rong Luo, Dezhi Wan, Lewei Xu, Lifang Deng, Lei Wu

**Affiliations:** 1 School of Public Health, Jiangxi Provincial Key Laboratory of Disease Prevention and Public Health, Jiangxi Medical College Nanchang University Nanchang China; 2 Jiangxi Provincial Center for Patriotic Health and Health Promotion Nanchang China; 3 The George Institute for Global Health Beijing China; 4 Chinese Center for Health Education Beijing China; 5 Wolfson Institute of Population Health, Barts and The London School of Medicine and Dentistry Queen Mary University of London London United Kingdom; 6 Jiangxi Association for Health Education and Tobacco Control Nanchang China

**Keywords:** school-based health education, EduSaltS, mobile health, salt reduction, cluster randomized trial

## Abstract

**Background:**

Globally, cardiovascular diseases are leading causes of mortality and disability, with hypertension being a major risk factor. Reducing salt intake and blood pressure are among the most cost-effective health promotion strategies. While mobile health (mHealth)– and school-based salt reduction interventions have proven effective in trials, their impact when scaled up in real-world contexts remains uncertain.

**Objective:**

We evaluated the effectiveness of the real-world implementation of an mHealth- and school-based health education scale-up program to reduce salt intake (EduSaltS [mHealth and school-based education program to reduce salt intake scaling up in China]).

**Methods:**

A parallel cluster randomized controlled trial was conducted from April 2022 to July 2023 across 20 schools in 2 districts and 2 counties within Ganzhou City, Jiangxi Province, China. Schools were randomized 1:1 to intervention or control groups within each district or county. One third-grade class per school and 26 students per class were randomly sampled. One parent, or alternative family member (aged 18-75 years, residing with the student), of each student was invited to join. The EduSaltS intervention, spanning over 1 academic year, incorporated both app-based health education courses and offline salt reduction activities, with participation monitored through the backend management system. The intervention’s effectiveness was assessed by comparing changes in salt intake and blood pressure between groups from baseline to 1-year follow-up using surveys, physical examination, and 24-hour urine tests.

**Results:**

Of 524 children (boys: n=288, 54.96%; age: mean 9.16, SD 0.35 years) and 524 adults (men: n=194, 37.02%; age: mean 40.99, SD 11.04 years) who completed the baseline assessments in 10 intervention and 10 control schools, 13 (2.48%) children and 47 (8.97%) adults were lost to follow-up. All schools and participants showed satisfactory intervention adherence. Measured differences in schoolchildren’s salt intake, systolic blood pressure, and diastolic blood pressure, between the intervention and control schools, were –0.24 g/day (95% CI –0.82 to 0.33), –0.68 mm Hg (95% CI –2.32 to 0.95), and –1.37 mm Hg (95% CI –2.79 to 0.06), respectively. For adults, the intervention group’s salt intake decreased from 9.0 (SE 0.2) g/day to 8.3 (SE 0.2) g/day post intervention. Adjusted changes in the intervention (vs control) group in salt intake, systolic blood pressure, and diastolic blood pressure were –1.06 g/day (95% CI –1.81 to –0.30), –2.26 mm Hg (95% CI –4.26 to –0.26), and –2.33 mm Hg (95% CI –3.84 to –0.82), respectively.

**Conclusions:**

The EduSaltS program, delivered through primary schools with a child-to-parent approach, was effective in reducing salt intake and controlling blood pressure in adults, but its effects on children were not significant. While promising for nationwide scaling, further improvements are needed to ensure its effectiveness in reducing salt intake among schoolchildren.

**Trial Registration:**

Chinese Clinical Trial Registry ChiCTR2400079893; https://tinyurl.com/4maz7dyv (retrospectively registered); Chinese Clinical Trial Registry ChiCTR2000039767; https://tinyurl.com/5n6hc4s2

## Introduction

Cardiovascular disease (CVD) stands as the foremost cause of death and disability worldwide, with hypertension identified as its major risk factor [1-4]. Substantial evidence indicates that excessive salt intake is a key contributor to elevated blood pressure and a significant risk factor for CVD [5-8]. Additionally, the incidence and progression of various noncommunicable diseases, such as chronic kidney diseases, are also linked to salt consumption [9]. Numerous systematic reviews and meta-analyses have demonstrated that reducing salt (sodium) intake can lower the risk of hypertension and CVD [10-13]. The World Health Organization considers reducing salt intake among populations as one of the most effective and economical strategies for health promotion.

China ranks as one of the countries with the highest salt intake, averaging about 11 g/day among adults [14], more than twice the World Health Organization’s recommended level of 5 g/day. Unlike developed countries where the majority of salt intake in the population originates from processed foods, approximately 80% of the salt consumption in the Chinese diet comes from the salt added during cooking [6]. Therefore, in addition to reducing sodium in food processing, lowering cooking salt is more crucial. To this end, the Chinese government has initiated policies such as the “Healthy China Initiative (2019-2030),” implementing a national dietary action for healthy living and advocating for “Three Reductions and Three Healthy Conditions,” with salt reduction being a primary focus. Meanwhile, a series of scientific studies [15-19] have also been conducted in China to create a supportive environment for salt reduction, optimize public health education and individualized interventions, and further refine comprehensive strategies for reducing salt intake.

**Figure 1:**
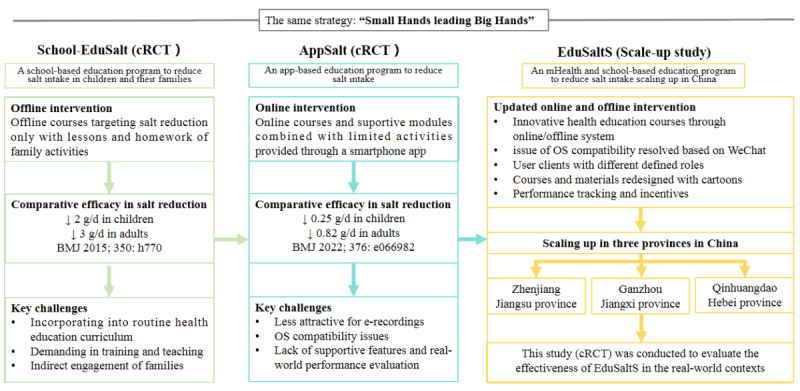
The evolution of studies from School-EduSalt, AppSalt to EduSaltS. cRCT: cluster randomized controlled trials; EduSaltS: mHealth and school-based education program to reduce salt intake scaling up in China; OS: operation system; School-EduSalt: school-based education program to reduce salt intake in children and their families.

Schools, as a critical place for health education, have been proven effective in addressing public health issues concerning smoking, obesity, and CVD [20-22]. The advent of modern information and mobile technologies has also further broadened the scope of health interventions [23,24]. A cluster randomized controlled trial (School-EduSalt [school-based education program to reduce salt intake in children and their families]) was conducted by our team to demonstrate that the “small hands leading big hands” strategy, in which schoolchildren encouraged their parents to collaboratively enhance their knowledge, attitudes, and practices related to salt reduction, effectively reduced salt intake among both children and adults [25]. However, its reliance on specialized offline courses and activities, which demanded considerable training for schoolteachers, limited large-scale implementation. To address this issue, a mobile health (mHealth) platform for salt reduction named AppSalt was developed to provide standardized app-based health education courses and parallel homework assignments for students and their parents. Subsequently, a cluster randomized controlled trial was conducted under close supervision and the results showed that AppSalt was also effective in lowering salt intake among adults [18]. Building on these research findings, an updated development and preliminary implementation study called the mHealth and school-based education program to reduce salt intake scaling up in China (EduSaltS [mHealth and school-based education program to reduce salt intake scaling up in China]) was launched in Ganzhou in Jiangxi province, Zhenjiang in Jiangsu province and Qinhuangdao in Hebei province, for the first time, to refine and scale up the intervention. Compared with the interventions in our previous studies, EduSaltS features a WeChat-based system (Tencent), cartoon-enhanced course materials, role-specific user interfaces, and blended online/offline health education, aiming for a routine yet innovative school-based approach [26]. The evolution of studies from School-EduSalt, AppSalt to EduSaltS can be seen in Figure 1.

Given the uncertainty surrounding the impact of EduSaltS in real-world settings, this study aims to evaluate its effectiveness in reducing salt intake through a cluster randomized controlled trial conducted in Ganzhou, Jiangxi Province in China.

## Methods

### Ethical Considerations

This study was approved by the Scientific Research Ethics Committee of the First Affiliated Hospital of Gannan Medical College (LLSC-2022011901) and was registered retrospectively at the Chinese Clinical Trial Registry (ChiCTR2400079893). This was due to its embedding in a prospectively registered scale-up intervention study (ChiCTR2000039767) and a lack of timely communication with the overall project team. Nevertheless, this study was identical to the protocol assessed by the Research Ethics Committee, which was prospectively approved on January 19, 2022. Permissions were obtained from the local education authorities and school headteachers. All participants taking part in the outcome assessments gave written informed consent. For children, participant assent and parental written consent were obtained. This report does not contain any identifying information or direct quotes. Due to the nature of the program, this study does not involve details of compensation.

### Study Design and Participants

The overall design and framework of EduSaltS program have been published elsewhere [26]. The study was implemented using a cluster randomized controlled trial design within scale-up, spanning across 20 public primary schools in Ganzhou City, Jiangxi Province, over a period from April 2022 to July 2023. The process of this study encompassed (1) determination of research sites, (2) execution of on-site baseline survey and clinical evaluations at the end of third grade, (3) allocation of the schools into 2 groups comprised of 10 schools in the intervention group and an equal number in the control group, (4) implementation of an integrative intervention and its subsequent promotion, conducted over the course of 2 semesters, and (5) undertaking of effect evaluation after intervention at the end of fourth grade.

EduSaltS was implemented in 100 scale-up schools across 9 subordinate districts and counties in Ganzhou city, mobilizing all third-grade students and their parents to participate in a year-long health education course and activities focused on salt reduction. In this context, to assess the effectiveness of the intervention, we first contacted the local education authority through the local Health Commission to gain their opinion, support, and approval for the study. After that, considering the potential diversity between urban and rural areas, 2 districts (Zhanggong and Nankang) and 2 counties (Xinfeng and Yudu) close to the city center were selected as the representative sites for the effectiveness assessments following the principle of proximity [27], which can help minimize the influence of geographical and environmental factors, before the commencement of EduSaltS. The selection of schools involved choosing a total of 20 schools from these 4 areas that were willing to participate in the trial, with an even number of schools from each district or county. These schools were then divided into the intervention or control group within each district or county in a 1:1 ratio. The 10 schools in the intervention group were included in the 100 scale-up schools participating in EduSaltS and received the unified interventions, while the control group was not exposed to the intervention and only received routine health education courses, including physical and mental health as all school in the city, but not covering salt reduction. Details of the scale-up schools participating in EduSaltS and the schools in the trial across different districts and counties are provided in Table S1 in Multimedia Appendix 1.

In each selected school, due to the substantial workload and costs associated with conducting the assessments, a simple random sampling method was used. This involved randomly selecting one third-grade class from each school, followed by the random selection of 26 students from the chosen class to serve as survey participants. Subsequently, one parent of each participating child was recruited. At this stage, invitations and explanatory materials were distributed to parents through the class teacher. If a parent was unavailable, a family member residing with the child, such as a grandparent, was eligible for selection as an alternative. To mitigate bias arising from sex imbalance, preference was given to selecting an adult of the opposite sex to the student whenever possible. In total, this study planned to recruit 520 students and an equivalent number of adults. The baseline survey and clinical evaluations were completed before the schools were allocated, and the follow-up assessments were conducted after the intervention had concluded.

Inclusion criteria were as follows: (1) willingness to participate in follow-up, (2) no transfer plans during the salt reduction intervention period for students, (3) ages of participating adults being older than 18 years and younger than 75 years, and (4) being able to provide regular feedback and complete outcome measurements. Exclusion criteria included: (1) children with medical conditions or other reasons precluding participation in the assessments or intervention activities, (2) individuals explicitly unable to complete follow-up, and (3) inability or refusal to collect 24-hour urine samples.

### Randomization and Masking

Schools in each district or county were randomly assigned to the intervention or control group (1:1) using a stratified method of random digits by researchers from Jiangxi Provincial Center for Patriotic Health and Health Promotion with the support of the local bureau of education. This ensured a balanced and comparable distribution of schools in 2 groups across districts and counties. Additionally, after the random allocation into intervention and control groups, a comparison of the geographic distribution of schools was conducted to prevent potential contamination by ensuring no close proximity between schools in different groups. The final grouping of schools is detailed in Table S2 in Multimedia Appendix 1.

Due to the specificity of the intervention, it was imperative to explicitly inform the intervention group of the relevant details. Therefore, blinding was not feasible for the intervention implementers. However, blinding was employed for data collectors. Following rigorous training, investigators from hospitals and Centers for Disease Prevention and Control, without knowing the group allocations, collected baseline and follow-up data. The technicians who performed urinary electrolyte measurements (KingMed Diagnostics) were unaware of specific groups for individual samples.

### Procedure

After selecting the schools and classes for the study, communication with school headteachers and class teachers was initiated. Class teachers were responsible for distributing letters of project introduction and informed consent forms to students and their parents (or guardians), thereby confirming the specific student and parent participants and facilitating the on-site baseline survey and clinical evaluations. The content comprised (1) sociodemographic characteristics (eg, age, sex, education level, and relationship to the participating children) and health behaviors (eg, smoking, alcohol consumption, and physical exercise); (2) measurements including height, weight, and blood pressure, which were conducted using standardized tools by trained staff from the Centers for Disease Control and Prevention and township health centers; and (3) a single 24-hour urine collection conducted on a weekday at the same time for both children and adults. The 24-hour urine collection was used to evaluate urinary sodium excretion, which is the most accurate method to estimate salt intake. All participants were provided with sufficient containers to collect their urine continuously over a 24-hour weekday period, ensuring a total collection time of 20-28 hours. After measuring the total urine volume, the thoroughly mixed sample was analyzed for sodium concentration, which was then multiplied by the total volume (corrected for any minor deviations from 24 hours) to calculate the 24-hour urinary sodium excretion. Salt intake was derived by multiplying the 24-hour urinary sodium excretion by 23 (atomic weight of sodium) and 2.54 (the sodium-to-salt conversion factor). The follow-up investigation was conducted after 1 year of intervention, using the same method.

### Intervention

The specific framework, development process, features, and intervention protocol of EduSaltS have been detailed in a previously published paper [26]. The intervention, which spanned 1 academic year, incorporated app-based courses, offline activities, and the establishment of a supportive salt-reduction environment. Quality control was mainly implemented by teachers following standardized training, with guidance from our research team, targeting all fourth-grade students and their families in intervention schools. The concise summary of interventions of EduSaltS is provided in Table S3 in Multimedia Appendix 1, and a detailed demonstration of specific applications and web interfaces can also be seen in our published paper [26].

The online component of this intervention predominantly revolved around the WeChat miniprogram named “Health Cloud Classroom”, delivering a curriculum of salt reduction. In alignment with the “Health Education Guidelines for Primary and Secondary Schools” stipulated by the Ministry of Education of China, the miniprogram disseminated 1 health lesson weekly and 10 lessons for 1 semester. Each video lesson, approximately 10 minutes in duration, was designed for students to watch at home with their family members, followed by a set of 5-10 quiz questions to enhance learning interest and assess comprehension. The curriculum encompassed eight compulsory lessons on salt reduction (covering the dangers of excessive salt intake, misconceptions, and practical techniques about salt reduction) and 12 elective health lessons (such as eye protection, nutritional diet, and influenza prevention). Class teachers had responsibilities for organizing students and their parents or grandparents to use the “Health Cloud Classroom” for course learning. They also used a teacher-oriented miniprogram named “Health Salt Management” (a WeChat app designed for monitoring, managing, and evaluating) to stay informed about the status of course learning and homework completion of all students and their families in each class, ensuring timely adherence to requirements of the curriculum. Each term, students were expected to complete 10 lessons. Moreover, “Health Cloud Classroom” features additionally optional modules designed to facilitate the increase of low-sodium knowledge and encourage corresponding behavioral changes, such as healthy food selection (explanations of various food nutrients), interactive quizzes on salt-related knowledge for entertainment, and routine assessments of sodium intake levels through self-administered dietary records. These modules were optional, and families were free to choose whether or not to use them without any monitoring of their use. Materials uploaded in this miniprogram for online study, such as cartoons, PowerPoint, and posters, could also be used for offline activities.

The offline component primarily involved salt reduction interactive activities. Integrating existing educational frameworks like health education classes, science practice classes, and themed class meetings, each term featured at least 4 lectures and themed activities concerning salt reduction. Class teachers could use videos, posters, and leaflets provided by “Health Cloud Classroom” to impart knowledge about salt reduction to students. Children were expected to complete related homework and convey salt reduction knowledge, methods, and skills to their family members after class, persuading the person cooking at home to reduce salt use and improve dietary behavior. Additionally, the program includes the construction of an environment of lower sodium and health education regarding reduced-salt school meals, such as displaying salt reduction posters across the campus, broadcasting a series of salt reduction short films and radio dramas within schools, and providing self-learning materials and training once per semester for cafeteria chefs and catering providers to lower the salt content in students’ meals.

To render the evaluation of the performance of the process more scientifically effective, a web-based miniprogram in WeChat named “EduSaltS Manager,” with regard to backend management and quality control, was developed specifically for researchers and school teachers. This program aided teachers in knowing and overseeing the participation of families and in submitting records related to project implementation. Researchers could also access relevant data through this backend system, regularly monitoring the progress and compliance in participants or specific schools.

Students in the control group received routine health education courses, occasionally delivered by physical education teachers, with no specific content on salt reduction and no family engagement. Due to the citywide publicity of EduSaltS, schools in the control group might have been aware of this project. However, as families in the control group were not provided with accounts to access the intervention miniprogram, there was little risk of contamination.

### Outcomes

The primary outcome of this study was the difference between the 2 groups in the change in salt intake among students and adults, before and after the intervention, assessed through 24-hour urinary sodium. The secondary outcome was the change in blood pressure. All measurements and data collections were conducted once at baseline and after completion of the intervention, with consistent survey processes and methods of data collection at each time point and across both groups.

The method for 24-hour urine collection was the same as previous studies, using an electronic data collection system [14]. Tests of all samples were uniformly conducted by a central laboratory (KingMed Diagnostics), with the testing parameters including urinary sodium, potassium, and creatinine concentrations. Urinary volume and creatinine concentration were used to assess the completeness of the 24-hour urine collection. The concentration of urinary sodium and potassium was used to calculate the participants’ salt and potassium intake, as well as the sodium-potassium ratio.

Blood pressure measurements were conducted using calibrated Omron electronic sphygmomanometers. After a 10-minute rest in a seated position, trained physicians measured the blood pressure of the right arm of the participants. Blood pressure was measured 3 times for each individual, with at least a 1-minute interval between measurements. The average of the last 2 readings was used for statistical analysis.

This study employed an electronic information platform to support data collection, allowing for the recording of the evidence of key survey indicators through audio recording, video recording, and photography, which standardized the collection of diverse data information.

### Sample Size Calculation

A mixed linear model was planned to be applied to analyze changes in effect sizes, with alterations in salt intake (calculated from 24-hour urinary sodium excretion) serving as the primary outcome variable. In accordance with previous research [25], a reduction of 1 g/day of salt intake was set as the target change in effect size, with an SD of 2.15 and an intraclass correlation coefficient of 0.01. Setting α at 0.05, 1-β at 0.9, and considering a 10% loss to follow-up rate, the sample size calculation performed using PASS software indicated that 520 students and 520 parents (cluster size of 26) across 10 intervention and 10 control schools would satisfy the power of the test.

### Statistical Analysis

The effectiveness of the intervention was evaluated through an intention-to-treat analysis without imputation due to the very low drop-out rates (13/524, 2.48% for children and 47/524, 8.97% for adults). All students and their family members who completed the baseline assessments were analyzed according to the initial group allocation of their respective schools. Taking into account the hierarchical structure of the data, with 2 measurements (baseline and 2 semesters) for each participant and participants nested within each school, mixed linear models with random intercepts and fixed effect variables (eg, time, group, time×group, and covariates) were applied to analyze the effects of intervention. The time×group interaction term indicated the differential change by groups from baseline to the end of the trial. Potential confounding variables adjusted in the model included district/county areas, age, sex, BMI (body weight in students), education level (the adults’ level substituted for their children), and physical exercise (coded as “yes” if someone engaged in moderate physical activities at least 3 times a week and more than 30 minutes each time). Additionally, when analyzing blood pressure changes, outdoor temperature was further included as a confounding factor. In the analysis of the effect on adults, familial relationships, smoking, and alcohol consumption (coding “occasional” and “regular” as “yes”) were added as additional adjustments.

The collection time of 24-hour urine samples was recorded in this study. The urine sample would be excluded if the collection time was less than 20 hours or more than 28 hours. If the duration of the collection was within 20-28 hours, the 24-hour urine volume was calculated as: urine volume (mL per 24 hours) = urine volume (mL) / duration of urine collection (hours) × 24 hours. This adjustment, in conjunction with urinary concentration of sodium and potassium, was used for the calculation of individual salt intake and other related indicators. Urine samples were also considered incomplete and excluded from the main analysis if 24-hour urine volume was <500 mL in adults; 24-hour urinary creatinine was <4.0 mmol or <6.0 mmol in women or men, respectively; 24-hour urine volume was <300 mL in children; or 24-hour urinary creatinine was lower than the fifth centile (<2.27 mmol for girls and <2.43 mmol for boys).

Sensitivity analysis and subgroup analysis were carried out in this study. In the sensitivity analysis, possibly incomplete 24-hour urine collections, assessed by 24-hour urinary volume and creatinine excretion, were included in the analysis based on the intention-to-treat approach. In the subgroup analysis, for students, the effects of intervention across different subgroups based on district/county areas, sex, and education levels of their familial participants were analyzed. For adults, additional analyses of subgroups were performed, which included age groups, education levels, blood pressure status (hypertension was defined as systolic blood pressure [SBP] ≥140 mm Hg or diastolic blood pressure [DBP] ≥90 mm Hg, or self-reported hypertension), smoking, weight status (BMI<24.0 or not), and relationship with their children.

Quantitative data were statistically described using means and SDs, while categorical data were presented as frequencies and percentages. Statistical analyses were executed using SAS (version 9.4; SAS Institute Inc), with a *P* value of <.05 considered statistically significant.

## Results

### Characteristics of Participants

**Figure 2:**
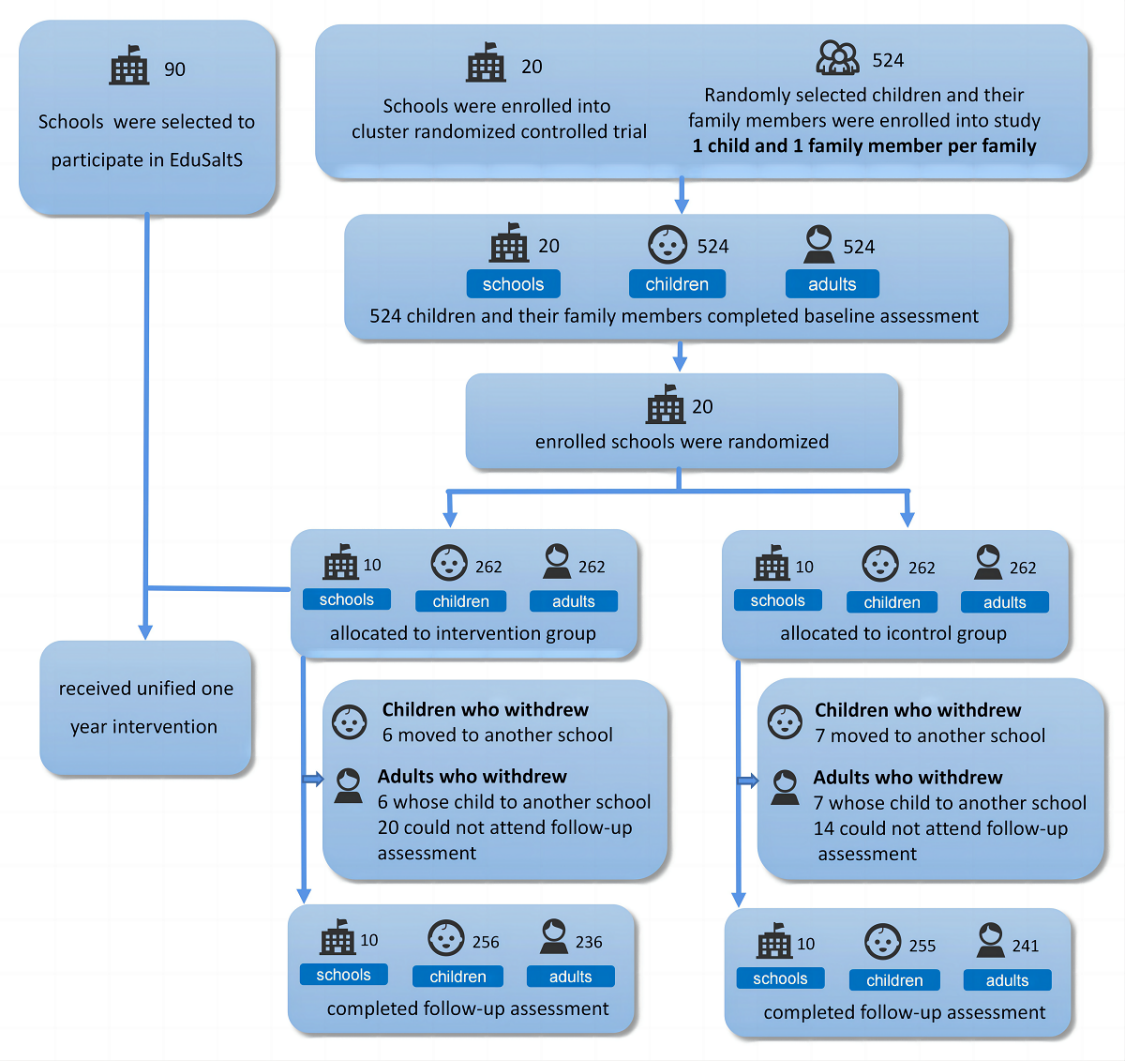
Research process of trial within scale-up.

Twenty schools across 4 districts or counties in Ganzhou City in southeastern China were recruited in this research. Adhering to the specified inclusion and exclusion criteria, a total of 524 families (4 families, beyond our initial expectations, from different schools were enthusiastic about this project, so we enrolled them as well) participated in the study and completed the baseline survey and clinical evaluations. Following randomization and 2 semesters of intervention, the loss to follow-up was 2.48% (13/524) for students and 8.97% (47/524) for adults, due to either the students having moved to another school or adults being unable to attend subsequent assessments, as detailed in Figure 2. Baseline characteristics of those lost to follow-up did not significantly differ from those who completed the study and the loss to follow-up was completely random (*P*>.05 for the Little test for both children and adults). In all families of the intervention and control groups who completed the baseline assessments, the average ages of the students were 9.10 (SD 0.35) and 9.22 (SD 0.34), respectively, while the average ages of adults were 40.96 (SD 11.07) and 41.01 (SD 11.03). Each of the intervention and control groups had 20 adults who self-reported having hypertension, and these individuals were confirmed to be on medication at the time of blood pressure measurement. Demographic and general behavioral characteristics of these 2 randomized groups were basically consistent and well-balanced, as detailed in Table 1.

**Table 1:** Basic characteristics at baseline.

Characteristics			Intervention	Control	Total
**Cluster level**					
	**Number of schools, by location, n (%)**				
		Zhanggong district	4 (40.00)	4 (40.00)	8 (40.00)
		Nankang district	3 (30.00)	3 (30.00)	6 (30.00)
		Xinfeng county	2 (20.00)	2 (20.00)	4 (20.00)
		Yudu county	1 (10.00)	1 (10.00)	2 (10.00)
	**Meal provision, n (%)**				
		Yes	6 (60.00)	6 (60.00)	12 (60.00)
		No	4 (40.00)	4 (40.00)	8 (40.00)
	Number of families, n		262	262	524
	Outdoor temperature (℃), mean (SD)		23.02 (1.68)	24.81 (2.21)	23.93 (2.16)
**Children level**					
	**Sex, n (%)**				
		Boys	142 (54.20)	146 (55.73)	288 (54.96)
		Girls	120 (45.80)	116 (44.27)	236 (45.04)
	Age (years), mean (SD)		9.10 (0.35)	9.22 (0.34)	9.16 (0.35)
	Weight (kg), mean (SD)		28.46 (5.95)	29.30 (6.70)	28.88 (6.34)
	BMI (kg/m^2^), mean (SD)		15.93 (2.34)	15.81 (2.79)	15.87 (2.57)
	**Physical activity, n (%)**				
		Yes	91 (34.73)	96 (36.64)	187 (35.69)
		No	171 (65.27)	166 (63.36)	337 (64.31)
**Adult level**					
	**Sex, n (%)**				
		Male	97 (37.02)	97 (37.02)	194 (37.02)
		Female	165 (62.98)	165 (62.98)	330 (62.98)
	Age (years), mean (SD)		40.96 (11.07)	41.01 (11.03)	40.99 (11.04)
	Weight, mean (SD)		59.98 (10.48)	59.49 (10.02)	59.74 (10.24)
	BMI, mean (SD)		23.76 (3.26)	23.23 (3.20)	23.50 (3.24)
	**Relationship with children, n (%)**				
		Parents	207 (79.01)	217 (82.82)	424 (80.92)
		Grandparents	50 (19.08)	43 (16.41)	93 (17.75)
		Other	5 (1.91)	2 (0.76)	7 (1.34)
	**Physical activity, n (%)**				
		Yes	98 (37.40)	88 (33.59)	186 (35.50)
		No	164 (62.60)	174 (66.41)	338 (64.50)
	**Education, n (%)**				
		≤Primary school	50 (19.08)	39 (14.89)	89 (16.98)
		Secondary education	117 (44.66)	106 (40.46)	223 (42.56)
		High school education	51 (19.47)	57 (21.76)	108 (20.61)
		College or above	44 (16.79)	60 (22.90)	104 (19.85)
	**Smoking, n (%)**				
		Yes	52 (19.85)	49 (18.70)	101 (19.27)
		No	210 (80.15)	213 (81.30)	423 (80.73)
	**Alcohol drinking, n (%)**				
		Never	158 (60.31)	151 (57.63)	309 (58.97)
		Occasional	96 (36.64)	97 (37.02)	193 (36.83)
		Regular	8 (3.05)	14 (5.34)	22 (4.20)
	**Self-reported hypertension, n (%)**				
		Yes	20 (7.63)	20 (7.63)	40 (6.62)
		No	242 (92.37)	242 (92.37)	484 (92.37)

### Evaluation of the Intervention

During the process of intervention implementation, all schools in the intervention group systematically cultivated an environment conducive to low-sodium practices in accordance with the prescribed program. Health education lessons incorporating salt reduction content, which were required to be conducted at least once every 2 weeks, were effectively implemented across all participating schools, offline activities were also carried out successfully. Furthermore, low-sodium materials provided by our research team, including videos, PowerPoint, posters, and pamphlets, were integrated into various educational activities such as health education courses, lectures, as well as thematic class meetings. In addition to these initiatives, we distributed low-sodium picture books and stationery to all families in order to boost the adherence of the participants, garnering a positive reception from students. All families of the intervention group registered and actively engaged with the WeChat-based “Health Cloud Classroom” miniprogram. Timely dissemination of app-based courses was ensured under the collective guidance of researchers and teachers. Finally, 84.7% (222/262) families successfully fulfilled the requirements of the app-based curriculum pertaining to low-sodium education in the “Health Cloud Classroom.” Overall, adherence to the intervention was satisfactory and the process evaluation of EduSaltS could be found in the published paper [28].

Table 2 presents the measurements and changes in salt intake, blood pressure, urinary sodium, potassium, and sodium-potassium ratio for the intervention and control groups. During the study of 2 semesters, there was a slight upward trend in salt intake and SBP among students. The salt intake of students was 5.4 (SE 0.2) g/day and 5.5 (SE 0.2) g/day in the intervention and control group at baseline, and it increased by 0.41 g/day (95% CI 0.01-0.82) and 0.61 g/day (95% CI 0.21-1.02) respectively after 2 semesters. The mean effect comparing the intervention group with the control group was –0.24 g/day (95% CI –0.82 to 0.33) after adjustment for covariates. Similarly, the blood pressure of students showed the same trend. The adjusted mean difference in the change of SBP and DBP for students between intervention and control groups was –0.68 mm Hg (95% CI –2.32 to 0.95) and –1.37 mm Hg (95% CI –2.79 to 0.06), respectively.

For adults, the intervention group showed a reduction in salt intake (g/day) and urinary sodium (mmol/24 hours), while the control group showed a slight increase, resulting in a significant difference. The salt intake in the intervention group decreased from 9.0 (SE 0.2) g/day to 8.3 (SE 0.2) g/day during the study, a change of –0.79 g/day (95% CI –1.33 to –0.25). In contrast, the control group exhibited a slight increase of 0.26 g/day (95% CI –0.27 to 0.80), from 9.1 (SE 0.2) g/day to 9.3 (SE 0.2) g/day. After adjusting for confounding factors, the mean intervention effect between the 2 groups was –1.06 g/day (95% CI –1.81 to –0.30), which was statistically significant. Regarding blood pressure, there were slight changes in the intervention group, whereas the control group showed a marked increase. The adjusted intervention effect, comparing the intervention group with the control group, was –2.26 mm Hg (95% CI –4.26 to –0.26) for SBP and –2.33 mm Hg (95% CI –3.84 to –0.82) for DBP. Further details are available in Table 2.

Table 3 shows the results of the sensitivity analysis. The adjusted mean effects comparing the intervention group with the control group on salt intake in children (–0.27 g/day, 95% CI –0.85 to 0.30) and in adults (–1.01 g/day, 95% CI –1.75 to –0.26) were similar to the main results when potentially incomplete 24-hour urine collections were included.

In the analysis of subgroups, as shown in Table S4 in Multimedia Appendix 1, no significant difference was seen among any of the interactions of subgroups with the intervention effect on salt intake for both children and adults. Relatively, younger adults (age<40 years), males, nonsmokers, being overweight (BMI≥24), and participants who were parents appeared to experience significantly greater effects of intervention. However, these outcomes may lack robustness due to the small sample size. Detailed results of the analysis of subgroups for SBP and DBP can be found in Tables S5 and S6 in Multimedia Appendix 1.

**Table 2:** Salt intake (g/24h, as calculated from 24-hour urinary sodium excretion), blood pressure, and other 24-hour urinary measurements based on intention-to-treat analysis.

		Intervention group					Control group					Adjusted difference in change^a^	
		Number^b^	Baseline, mean (SE)	12 months, mean (SE)	Change^c^, (95% CI)	Number^b^		Baseline, mean (SE)	12 months, mean (SE)	Change^c^, (95% CI)	Intervention vs control, (95% CI)		*P* value
**Children**													
	Salt intake	261	5.4 (0.2)	5.7 (0.2)	0.41 (0.01 to 0.82)	261		5.5 (0.2)	6.1 (0.2)	0.61 (0.21 to 1.02)	–0.24 (–0.82 to 0.33)		.41
	SBP^d^	262	94.6 (0.6)	97.2 (0.6)	2.65 (1.50 to 3.80)	262		94.8 (0.6)	98.0 (0.6)	3.22 (2.07 to 4.37)	–0.68 (–2.32 to 0.95)		.41
	DBP^d^	262	60.5 (0.4)	60.6 (0.4)	0.09 (–0.90 to 1.09)	262		58.9 (0.5)	60.4 (0.5)	1.45 (0.45 to 2.45)	–1.37 (–2.79 to 0.06)		.06
	Na^e^	261	91.8 (2.6)	98.2 (2.8)	7.06 (0.21 to 13.93)	261		93.4 (3.1)	104.1 (2.9)	10.50 (3.57 to 17.42)	–4.15 (–13.94 to 5.65)		.41
	K^e^	261	21.6 (0.5)	23.8 (0.7)	2.40 (0.60 to 4.19)	261		23.9 (0.7)	25.4 (0.9)	1.37 (–0.45 to 3.18)	1.01 (–1.54 to 3.57)		.44
	Na/K^e^	261	4.6 (0.1)	4.7 (0.2)	0.10 (–0.24 to 0.44)	261		4.5 (0.2)	4.8 (0.1)	0.32 (–0.03 to 0.66)	–0.26 (–0.75 to 0.22)		.29
**Adults**													
	Salt intake	256	9.0 (0.2)	8.3 (0.2)	–0.79 (–1.33 to –0.25)	257		9.1 (0.2)	9.3 (0.2)	0.26 (–0.27 to 0.80)	–1.06 (–1.81 to –0.30)		.007
	SBP^d^	262	112.9 (1.0)	112.9 (1.0)	0.43 (–1.01 to 1.87)	262		111.5 (1.1)	113.8 (1.1)	2.89 (1.47 to 4.32)	–2.26 (–4.26 to –0.26)		.03
	DBP^d^	262	73.0 (0.7)	72.4 (0.7)	–0.47 (–1.57 to 0.63)	262		71.5 (0.7)	73.3 (0.7)	2.05 (0.96 to 3.14)	–2.33 (–3.84 to –0.82)		.003
	Na^e^	256	154.3 (4.2)	141.4 (4.1)	–13.47 (–22.76 to –4.19)	257		155.3 (4.0)	159.1 (4.1)	4.50 (–4.61 to 13.61)	–18.04 (–31.02 to –5.05)		.007
	K^e^	256	31.7 (0.7)	33.0 (0.9)	1.26 (–0.83 to 3.34)	257		33.9 (0.9)	33.1 (1.0)	–0.67 (–2.72 to 1.38)	2.10 (–0.83 to 5.03)		.16
	Na/K^e^	256	5.2 (0.1)	4.8 (0.2)	–0.42 (–0.79 to –0.05)	257		5.2 (0.2)	5.3 (0.2)	0.19 (–0.17 to 0.55)	–0.64 (–1.15 to –0.13)		.01

^a^Comparison between intervention and control groups in the changes from baseline to 12-month follow-up. Positive values=the intervention group had a greater increase or less decrease from baseline to 12-month follow-up than the control group; negative values=the intervention group had a greater decrease or smaller increase from baseline to 12-month follow-up than the control group. Results were adjusted for age, sex, BMI (body weight in children instead), district or county, physical exercise, and education level (the education level of the familial participant in children instead). In adults, additional adjustments were made for smoking, alcohol consumption, and relationship with the child. Blood pressure values were further adjusted for outdoor temperature.

^b^Number of participants included in the analysis.

^c^Comparison of the means between baseline and 12-month follow-up. Positive values=increases from baseline to 12-month follow-up; negative values=reductions from baseline to 12-month follow-up. Results were obtained from a mixed linear model taking into account the hierarchical structure of data.

^d^Systolic blood pressure (SBP) and diastolic blood pressure (DBP) are expressed in mm Hg (millimeters of mercury).

^e^Na and K represent the 24-hour urinary sodium excretion and urinary potassium excretion, respectively, both expressed in mmol/L. The Na/K ratio refers to the urinary sodium-to-potassium ratio.

**Table 3:** Salt intake (g/24h, as calculated from 24-hour urinary sodium excretion) and other 24-hour urinary measurements based on sensitivity analysis.

			Intervention group									Control group									Adjusted difference in change^a^		
			Number^b^		Baseline, mean (SE)		12 months, mean (SE)		Change^c^, (95% CI)		Number^b^			Baseline, mean (SE)		12 months, mean (SE)		Change^c^, (95% CI)		Intervention vs Control, (95% CI)			*P* value
**Children**																							
	Salt intake	262		5.2 (0.1)		5.6 (0.2)		0.36 (–0.04 to 0.76)		262			5.3 (0.2)		5.9 (0.2)		0.59 (0.19 to 1.00)		–0.27 (–0.85 to 0.30)			.35	
	Na^d^	262		89.3 (2.5)		95.4 (2.8)		6.15 (–0.75 to 13.05)		262			90.2 (3)		100.2 (2.9)		10.12 (3.21 to 17.02)		–4.69 (–14.52 to 5.13)			.35	
	K^d^	262		21.0 (0.5)		23.2 (0.7)		2.21 (0.39 to 4.02)		262			23.0 (0.7)		24.6 (0.9)		1.56 (–0.26 to 3.38)		0.67 (–1.91 to 3.24)			.61	
	Na/K^d^	262		4.6 (0.1)		4.7 (0.1)		0.07 (–0.26 to 0.41)		262			4.5 (0.2)		4.7 (0.1)		0.28 (–0.05 to 0.62)		–0.26 (–0.73 to 0.21)			.28	
**Adults**																							
	Salt intake	262		8.8 (0.2)		7.9 (0.2)		–0.90 (–1.43 to –0.37)		262			9.0 (0.2)		9.1 (0.2)		0.15 (–0.37 to 0.68)		–1.01 (–1.75 to –0.26)			.008	
	Na^d^	262		151.1 (4.2)		135.7 (4.0)		–15.46 (–24.51 to –6.40)		262			153.0 (4.0)		155.6 (4.0)		2.60 (–6.37 to 11.57)		–17.22 (–29.92 to –4.53)			.008	
	K^d^	262		30.9 (0.7)		31.3 (0.9)		0.40 (–1.64 to 2.44)		262			33.2 (0.9)		32.0 (1.0)		–1.24 (–3.26 to 0.78)		1.94 (–0.93 to 4.81)			.18	
	Na/K^d^	262		5.2 (0.1)		4.8 (0.2)		–0.41 (–0.76 to –0.07)		262			5.3 (0.2)		5.4 (0.2)		0.21 (–0.14 to 0.56)		–0.64 (–1.13 to –0.15)			.01	

^a^Comparison between intervention and control groups in the changes from baseline to 12-month follow-up. Positive values=the intervention group had a greater increase or less decrease from baseline to 12-month follow-up than the control group; negative values=the intervention group had a greater decrease or smaller increase from baseline to 12-month follow-up than the control group. Results were adjusted for age, sex, BMI (body weight in children instead), district or county, physical exercise, and education level (the education level of the familial participant in children instead). In adults, additional adjustments were made for smoking, alcohol consumption, and relationship with the child. Blood pressure values were further adjusted for outdoor temperature.

^b^Number of participants included in the analysis.

^c^Comparison of the means between baseline and 12-month follow-up. Positive values=increases from baseline to 12-month follow-up; negative values=reductions from baseline to 12-month follow-up. Results were obtained from a mixed linear model taking into account of the hierarchical structure of data.

^d^Na and K represent the 24-hour urinary sodium excretion and urinary potassium excretion, respectively, both expressed in mmol/L. The Na/K ratio refers to the urinary sodium-to-potassium ratio.

## Discussion

### Principal Results

This study deployed an mHealth- and school-based health education program for salt reduction (EduSaltS), a hybrid of online and offline methods, tailored for integration into school health education and promotion initiatives, which used a “small hands leading big hands” approach to cultivate awareness and practices of salt reduction among students and their families. It was the first time to evaluate the effectiveness of this scale-up program for salt reduction using an embedded randomized controlled trial.

Currently, salt reduction and hypertension prevention efforts among the general population in China are primarily carried out through public health education activities, such as the “Three Reductions and Three Health” campaign, and the national basic public health services. Although these measures play a pivotal role in salt reduction and hypertension prevention strategies in China, it is challenging for such efforts to reach or impact every household. The EduSaltS approach, however, has the potential to balance both broad coverage and effectiveness, which can serve as a good complement to existing strategies. Our research achieved high levels of participation and cooperation over an academic year, with follow-up rates of students surpassing 95% and adults over 90%. The strength of the EduSaltS intervention lies in its comprehensive strategy that integrates traditional school-based health education with the modern advantages of mHealth technology. This approach was not commonly observed in other school-based cardiovascular interventions, which often focused exclusively on students, for example, interventions such as the Active Smarter Kids physical activity intervention concentrated on increasing physical activity to improve cardiovascular health markers [29]. Similarly, the Salud Integral Program also focused on in-person interventions without integrating mHealth strategies that could have enhanced its overall impact [30]. Moreover, the EduSaltS intervention extended its impact beyond the individual by incorporating the “small hands leading big hands” approach, which encourages collaborative efforts within families to adopt low-salt dietary habits. In contrast, the interventions such as using SaltSwitch (The George Institute for Global Health) smartphone app [31] and WhatsApp (WhatsApp LLC) [32], while effective in their own right, focused more on direct educational outreach to individuals, without explicitly addressing family or community-level behavior change. In essence, the EduSaltS intervention’s combination of app-based engagement and offline activities, along with its emphasis on school and family involvement, positions it as a more holistic and potentially more effective strategy for reducing salt intake at both the individual and population levels.

Despite the relatively small and nonsignificant effect of the intervention on students’ salt intake, some effects of the intervention on both salt intake and blood pressure were observed among adults compared with the control group. The intervention period overlapped with a significant shift of the quarantine strategy of COVID-19 in China, from stringent isolation controls in 2022, limiting outings and social gatherings, to a relaxation of restrictions at the beginning of 2023, leading to an increase in dining out and leisure activities. From periods of home isolation to the resumption of regular social interactions, there may have been substantial changes in people’s dietary behaviors and work-life habits. In this context, the adult participants in the control group showed a nonsignificant rise in salt intake and an obvious increase in blood pressure, whereas the intervention group experienced a notable reduction in salt intake and a slight decrease in DBP, underscoring the effectiveness of the intervention among adults. Subgroup analysis indicated that the EduSaltS program seemed to be more effective among younger adults and those who are parents compared with grandparents. This result may be attributed to the familiarity of those populations with electronic devices and receptiveness to app-based information [33]. However, given the smaller sample size, the outcome of the subgroup analysis needs cautious interpretation.

The observed nonsignificant effects of the intervention in students may potentially be ascribed to a confluence of multifaceted factors. First of all, the salt intake among students is subject to a myriad of influences, including familial dietary patterns, the dining environment within and around the vicinity of schools, personal preferences, as well as levels of health consciousness. During the implementation of EduSaltS in Ganzhou City, the primary focus was on the family dimension and the completion of app-based courses. However, implementation in the school context, particularly in school catering, was not thoroughly actualized. Concurrently, elementary students exhibit a predilection for high-sodium snacks and fast foods, with their dietary choices markedly swayed by peer influences and personal taste preferences, often at the expense of healthful considerations. Such tendencies are detrimental to achieving the anticipated effectiveness of the intervention. Second, the comprehensive intervention in this study largely relied on the WeChat miniprogram named “Health Cloud Classroom,” necessitating parental involvement using smartphones or tablets alongside their children. However, some parents, merely to comply with teachers’ requests, operated the program themselves, resulting in interventions that often affected only the parents without the students, hardly obtaining the desired outcome. Furthermore, the same content of health intervention may not be as comprehensively understood by students as by parents. This disparity in comprehension and a less holistic grasp of health concepts are likely to result in an inadequate acquisition of knowledge and a lack of behavioral change among students.

### Public Health Implications

It is noteworthy that, although the findings of this study resembled, to some extent, that of our earlier study [14], the intervention incorporated here was broader in scope, more diverse in format, and easier to implement. As a result, the intervention schools have successfully established a supportive environment for salt reduction, and there has been a high level of cooperation among teachers, students, and parents in completing both app-based courses and offline activities. Building upon the already developed health education materials and platforms of intervention, the overall execution was relatively uncomplicated, just requiring primarily efficacious organization by schools and weekly opportune reminders by class teachers following brief training. Additionally, the resources within the system could assist teachers in managing their existing required health education courses and activities more efficiently, thus reducing the need for substantial manpower and resources. Previous research has demonstrated that a reduction of 1 g/day in per capita salt intake could lead to a decrease of nearly 9 million cardiovascular events by 2030 [34]. Furthermore, based on a recent meta-analysis of blood pressure treatment trials [35], the prevented increase of 2.26 mm Hg in SBP, compared with the rise observed in the control group in our study, is projected to reduce the risk of stroke by 5.88% and ischemic heart disease by 3.62%, which would prevent nearly 231,400 strokes and about 126,700 ischaemic heart disease events per year in China if the program was scaled up across the country [36]. Consequently, the initiative of EduSaltS, with its notable practicality, may not only facilitate the enhancement of health education curricula and activities in schools but also significantly curtail the prevalence of CVD within the population, rendering it of substantial value for the guidance of health policies and public health.

### Limitations

This study still has some limitations. For instance, during the implementation of the intervention, many aspects required the use of smartphones or tablets, which were not readily accessible in less developed, remote mountain areas, and for students only living with grandparents. Despite the assurance of access to basic public health and health care services in these populations, the digital divide and limitations in school infrastructure may hinder the implementation and effectiveness of the intervention. Thus, we should consider how to promote this mHealth technology–based health education in impoverished and underdeveloped areas, which may serve as a good supplement for the prevention and treatment of hypertension in regions with relatively lower medical care standards. Additionally, a minority of parents perceived this intervention as increasing children’s screen time, potentially detrimental to their eyesight, thereby engendering a degree of resistance. Furthermore, the intervention for school chefs and food service providers was limited to self-learning materials and training once per semester, without monitoring the salt used in school meals. Recording and monitoring salt purchase and use had been suggested but not implemented due to various difficulties. These are what we aim to improve in the future. Subsequent research endeavors will further analyze its generalizability and attempt to evaluate the long-term effects of the intervention. Our team will also incorporate feedback from process evaluations to refine and better integrate the comprehensive intervention strategy into the construction of healthy schools, ultimately enhancing the health levels of students and their parents.

### Conclusion

Based on extensive and long-term exploratory efforts, an attractive, appropriate, and comprehensive intervention program for salt reduction (EduSaltS) was developed and implemented in China. Demonstrating favorable feasibility and efficacy, this program is potentially well-suited for nationwide implementation based on collaboration across multiple departments. The intervention, adopting a “small hands leading big hands” approach, effectively reduced the salt intake and blood pressure among adults, while its impact on students was not statistically significant. Future enhancements to the offline components of the intervention, such as improving salt reduction interventions in school catering, could further facilitate the development of low-salt dietary habits in schoolchildren.

## Appendix

Multimedia Appendix 1 Additional tables.

Multimedia Appendix 2 CONSORT-eHEALTH checklist (V 1.6.1).

## Abbreviations

CVD: cardiovascular disease
DBP: diastolic blood pressure
EduSaltS: mHealth and school-based education program to reduce salt intake scaling up in China
mHealth: mobile health
SBP: systolic blood pressure
School-EduSalt: school-based education program to reduce salt intake in children and their families

## Data Availability

The datasets used or analyzed during this study are available from the corresponding author on reasonable request.
