# Supplementary material for: Effectiveness of an mHealth- and School-Based Health Education Program for Salt Reduction (EduSaltS) in China: Cluster Randomized Controlled Trial Within Scale-Up
Source: J Med Internet Res. 2025 Mar 27;27:e60092. doi: 10.2196/60092 (PMC11986378; doi:10.2196/60092)
Supplement: Multimedia Appendix 1 [file jmir_v27i1e60092_app1.docx]

Table S1. The number of schools participating in EduSaltS and cluster randomaized control trail across different districts and counties.^a^

| District/County | Total number of directly administered by government and central public primary schools | Number of schools participating in EduSaltS | Intervention group^a^ | Control group |
| --- | --- | --- | --- | --- |
| Zhanggong District | 32 | 13 | 4 | 4 |
| Nankang District | 32 | 12 | 3 | 3 |
| Xinfeng County | 34 | 12 | 2 | 2 |
| Yudu County | 38 | 13 | 1 | 1 |
| Ganxian District | 37 | 10 | - | - |
| Xunwu County | 23 | 10 | - | - |
| Shicheng County | 22 | 10 | - | - |
| Longnan County | 25 | 10 | - | - |
| Shangyou County | 21 | 10 | - | - |

^a^Schools in the intervention group were parts of 100 schools in EduSaltS and received the unified interventions.

Table S2. Distribution of intervention and control groups in 20 public primary schools.

| District/County | Intervention Group Schools | Control Group Schools |
| --- | --- | --- |
| Zhanggong District | Tianzhu Mountain Primary School, Shahe Central Primary School,  Shashi Central Primary School, Zhangjiang Road Primary School | Baiyun Primary School,  Yangming Primary School,  Railway Primary School,  Second Primary School |
| Nankang District | Sixth Primary School,  Fushi Central Primary School,  Sanyi Central Primary School | Dongshan Central Primary School,  Ninth Primary School,  Longling Central Primary School |
| Xinfeng County | Xinfeng Third Primary School,  Hope Primary School | Xinfeng Fifth Primary School,  Xinfeng Ninth Primary School |
| Yudu County | Yudu Third Primary School | Changzhengyuan Red Army Primary School |

Table S3. Summary of Interventions and activities of EduSaltS.^a^

| Parts | | Contents |
| --- | --- | --- |
| **Online interventions** | |  |
|  | Health education course | Description: 20 cartoon-style health education lessons, including quizzes and practices, with each session lasting five minutes. Twelve lessons focus on routine health education, and eight specifically on salt reduction. All of these are based on the WeChat mini-program - “Health Cloud Classroom”.  Users/Places: Students and families. |
|  | Supportive features | Description: Offering a library of various educational materials suited for different populations and settings, includes a knowledge competition, family salt intake monitoring, FoodSwitch module for healthy food selection, canteen food evaluation, Q&A, and promotion mechanisms.  Users/Places: Students and families. |
| **Offline interventions** | |  |
|  | School environment building | Description: Focusing on creating a healthy school environment with clear requirements, data and material support, evidence collection, and performance evaluation.  Users/Places: Schools, classes, and canteens. |
|  | Interactive activities in schools | Description: Schools and classes are encouraged to organize various interactive activities to reinforce health education, including health knowledge competitions, class meetings, and parent meetings. Most activities are optional but come with a designated purpose and design.  Users/Places: Schools, classes, and families. |
| **Management and evaluation systems** | |  |
|  | App-Based management-'EduSaltS manager' | Description: User administration, online/offline monitoring and feedback, technical support, evidence collection for offline activities, and education materials downloading.  Users/Places: Schools and government administrators. |
|  | Web-based management | Description: Involving school recruitment, role setting, system setting, announcements of temporal notices and activities, evidence confirmation, performance summaries, and specific statistics management.  Users/Places: Government administrators. |

^a^Referred to the published paper concerning the implementation study [26].

Table S4. Salt intake (g/day) as measured by 24 hour urinary sodium excretion by subgroup.^a-e^

|  | | Intervention group | | | | Control group | | | | Adjusted difference in change^e^ | |
| --- | --- | --- | --- | --- | --- | --- | --- | --- | --- | --- | --- |
|  | | n^b^ | Baseline mean(SE) | 12 months mean(SE) | Change^d^ (95%CI) | n^b^ | Baseline  mean(SE) | 12 months  mean(SE) | Change^d^ (95%CI) | Intervention VS Control (95%CI) | *P* |
| **Children** | |  |  |  |  |  |  |  |  |  |  |
| **Gender** | |  |  |  |  |  |  |  |  |  |  |
|  | Boys | 141 | 5.8(0.2) | 6.0(0.2) | 0.26(-0.33 to 0.84) | 145 | 5.5(0.2) | 6.3(0.2) | 0.79(0.21 to 1.37) | -0.59(-1.41 to 0.24) | 0.16 |
|  | Girls | 120 | 4.9(0.2) | 5.4(0.2) | 0.61(0.07 to 1.15) | 116 | 5.4(0.3) | 5.8(0.2) | 0.39(-0.17 to 0.94) | 0.24(-0.53 to 1.02) | 0.54 |
| **Area** | |  |  |  |  |  |  |  |  |  |  |
|  | County | 78 | 5.7(0.3) | 5.7(0.3) | -0.04(-0.87 to 0.80) | 78 | 6.1(0.5) | 6.0(0.3) | -0.14(-1.02 to 0.73) | 0.02(-1.18 to 1.23) | 0.97 |
|  | District | 183 | 5.2(0.2) | 5.8(0.2) | 0.62(0.18 to 1.06) | 183 | 5.2(0.2) | 6.1(0.2) | 0.92(0.47 to 1.36) | -0.31(-0.94 to 0.32) | 0.33 |
| **Education level of the familial participant** | |  |  |  |  |  |  |  |  |  |  |
|  | ≤Primary school | 50 | 5.5(0.3) | 5.6(0.3) | 0.17(-0.92 to 1.26) | 39 | 6.2(0.6) | 6.5(0.5) | 0.25(-1.05 to 1.54) | -0.36(-2.10 to 1.39) | 0.68 |
|  | Secondary education | 116 | 5.3(0.2) | 6.0(0.3) | 0.72(0.12 to 1.31) | 105 | 5.4(0.3) | 6.0(0.3) | 0.55(-0.08 to 1.17) | 0.14(-0.72 to 1.00) | 0.75 |
|  | High school | 51 | 5.3(0.3) | 5.6(0.4) | 0.41(-0.37 to 1.19) | 57 | 5.0(0.4) | 6.2(0.4) | 1.24(0.51 to 1.98) | -0.79(-1.86 to 0.29) | 0.15 |
|  | College or above | 44 | 5.4(0.4) | 5.4(0.3) | 0.03(-0.98 to 1.05) | 60 | 5.5(0.3) | 5.9(0.3) | 0.33(-0.53 to 1.19) | -0.41(-1.75 to 0.94) | 0.55 |
| **Adults** | |  |  |  |  |  |  |  |  |  |  |
| **Age group** | |  |  |  |  |  |  |  |  |  |  |
|  | <40 | 166 | 9.2(0.3) | 8.5(0.3) | -0.78(-1.49 to -0.06) | 165 | 9.0(0.3) | 9.4(0.3) | 0.37(-0.34 to 1.07) | -1.24(-2.24 to -0.24) | 0.02 |
|  | ≥40 | 90 | 8.7(0.4) | 7.9(0.4) | -0.80(-1.62 to 0.01) | 92 | 9.2(0.3) | 9.2(0.4) | 0.07(-0.72 to 0.86) | -0.74(-1.87 to 0.39) | 0.20 |
| **Gender** | |  |  |  |  |  |  |  |  |  |  |
|  | Male | 92 | 9.5(0.5) | 8.8(0.4) | -0.75(-1.61 to 0.12) | 93 | 9.7(0.4) | 10.2(0.4) | 0.59(-0.25 to 1.44) | -1.33(-2.56 to -0.10) | 0.03 |
|  | Female | 164 | 8.8(0.3) | 8.0(0.3) | -0.82(-1.52 to -0.13) | 164 | 8.7(0.3) | 8.8(0.3) | 0.11(-0.58 to 0.79) | -1.02(-2.00 to -0.03) | 0.04 |
| **Area** | |  |  |  |  |  |  |  |  |  |  |
|  | County | 77 | 8.6(0.4) | 7.7(0.4) | -0.98(-1.92 to -0.04) | 78 | 8.6(0.5) | 8.8(0.4) | 0.39(-0.54 to 1.32) | -1.19(-2.56 to 0.15) | 0.08 |
|  | District | 179 | 9.2(0.3) | 8.5(0.3) | -0.70(-1.37 to -0.03) | 179 | 9.3(0.3) | 9.5(0.3) | 0.22(-0.43 to 0.88) | -0.89(-1.83 to 0.06) | 0.07 |
| **Education level** | |  |  |  |  |  |  |  |  |  |  |
|  | ≤Primary school | 50 | 8.7(0.5) | 8.6(0.5) | -0.15(-1.29 to 1.00 ) | 39 | 9.0(0.5) | 9.2(0.6) | 0.22(-1.12 to 1.56) | -0.42(-2.18 to 1.33) | 0.64 |
|  | Secondary education | 112 | 9.4(0.4) | 8.5(0.4) | -0.89(-1.79 to -0.00) | 104 | 8.9(0.4) | 9.5(0.4) | 0.63(-0.25 to 1.53) | -1.61(-2.87 to -0.34) | 0.01 |
|  | High school | 51 | 8.7(0.4) | 7.6(0.5) | -1.09( -2.22 to 0.05) | 56 | 9.4(0.5) | 9.4(0.5) | -0.01(-1.09 to 1.07) | -0.98(-2.59 to 0.62) | 0.23 |
|  | College or above | 43 | 8.9(0.7) | 7.9(0.6) | -0.96(-2.25 to 0.33) | 58 | 9.1(0.5) | 8.9(0.5) | -0.15(-1.24 to 0.94) | -0.98(-2.73 to 0.78) | 0.27 |
| **Blood pressure status** | |  |  |  |  |  |  |  |  |  |  |
|  | Normotensive | 207 | 8.8(0.3) | 8.1(0.3) | -0.71(-1.31 to -0.11) | 217 | 9.2(0.3) | 9.3(0.3) | 0.20(-0.38 to 0.77) | -0.97(-1.80 to -0.13) | 0.02 |
|  | Hypertensive | 49 | 9.9(0.7) | 8.8(0.5) | -1.11(-2.43 to 0.22) | 40 | 8.7(0.5) | 9.3(0.6) | 0.72(-0.81 to 2.06) | -1.59(-3.53 to 0.34) | 0.10 |
| **BMI** | |  |  |  |  |  |  |  |  |  |  |
|  | <24 | 132 | 8.2(0.3) | 7.8(0.3) | -0.59(-1.35 to 0.18) | 152 | 8.8(0.3) | 8.8(0.3) | 0.15(-0.54 to 0.83) | -0.71(-1.73 to 0.31) | 0.17 |
|  | ≥24 | 124 | 9.8(0.4) | 8.7(0.4) | -1.05(-1.84 to -0.27) | 105 | 9.5(0.3) | 10.0(0.4) | 0.43(-0.42 to 1.28) | -1.42(-2.58 to -0.27) | 0.02 |
| **Smoking** | |  |  |  |  |  |  |  |  |  |  |
|  | No | 204 | 8.8(0.3) | 8.1(0.3) | -0.66(-1.27 to -0.05) | 209 | 8.9(0.3) | 9.2(0.3) | 0.27(-0.32 to 0.86) | -0.91(-1.75 to -0.06) | 0.04 |
|  | Yes | 52 | 10.0(0.6) | 8.7(0.5) | -1.34(-2.54 to -0.14) | 49 | 9.8(0.6) | 10.0(0.6) | 0.28(-0.97 to 1.53) | -1.76(-3.53 to 0.01) | 0.05 |
| **Relationship** | |  |  |  |  |  |  |  |  |  |  |
|  | Parents | 202 | 9.3(0.3) | 8.5(0.3) | -0.82(-1.47 to -0.18) | 213 | 9.1(0.3) | 9.4(0.3) | 0.27(-0.35 to 0.88) | -1.13(-2.02 to -0.24) | 0.01 |
|  | Grandparents | 50 | 8.1(0.5) | 7.6(0.4) | -0.51(-1.42 to 0.40) | 42 | 8.8(0.5) | 8.9(0.5) | 0.24(-0.78 to 1.25) | -0.66(-2.06 to 0.73) | 0.35 |
|  | Other^c^ | 4 | - | - | - | 2 | - | - | - | - | - |

^a^No significance was found in any subgroup variable with time and group (subgroup variable×time×group).

^b^N denotes the number of participants included in the analysis.

^c^Not being analyzed for too small sample size.

^d^Comparison of the means between baseline and 12-month follow-up. Positive values=increases from baseline to 12-month follow-up; negative values=reductions from baseline to 12-month follow-up. Results were obtained from mixed linear model taking into account of the hierarchical structure of data.

^e^Comparison between intervention and control groups in the changes from baseline to 12-month follow-up. Positive values=the intervention group had a greater increase or less decrease from baseline to 12-month follow-up than the control group; negative values=the intervention group has a greater decrease or smaller increase from baseline to 12-month follow-up than the control group. Results were adjusted for age, gender, body mass index (body weight in children instead), district or county, physical exercise, and education level (the education level of the familial participant in children instead). In adults, additional adjustments were made for smoking, alcohol consumption, and relationship with the child. Blood pressure values were further adjusted for outdoor temperature.

Table S5. Systolic blood pressure (mm Hg) by subgroup.^a-e^

|  | | Intervention group | | | | Control group | | | | Adjusted difference in change^e^ | |
| --- | --- | --- | --- | --- | --- | --- | --- | --- | --- | --- | --- |
|  | | n^b^ | Baseline mean(SE) | 12 months  mean(SE) | Change^d^ (95%CI) | n^b^ | Baseline  mean(SE) | 12 months  mean(SE) | Change^d^(95%CI) | Intervention VS Control (95%CI) | *P* |
| **Children** | |  |  |  |  |  |  |  |  |  |  |
| **Gender** | |  |  |  |  |  |  |  |  |  |  |
|  | Boys | 142 | 94.8(0.8) | 97.8(0.8) | 3.03(1.42 to 4.64) | 146 | 95.6(0.8) | 98.7(0.8) | 3.11(1.52 to 4.70) | -0.38(-2.66 to 1.90) | 0.74 |
|  | Girls | 120 | 94.3(0.8) | 96.5(0.9) | 2.19(0.54 to 3.84) | 116 | 93.8(0.9) | 97.1(0.9) | 3.37(1.70 to 5.04) | -1.14(-3.49 to 1.21) | 0.34 |
| **Area** | |  |  |  |  |  |  |  |  |  |  |
|  | County | 78 | 94.3(1.0) | 95.9(1.1) | 1.69(-0.30 to 3.68) | 78 | 93.3(1.1) | 96.8(1.0) | 3.71(1.72 to 5.70) | -2.31(-5.21 to 0.59) | 0.12 |
|  | District | 184 | 94.7(0.7) | 97.8(0.7) | 3.06(1.65 to 4.48) | 184 | 95.4(0.7) | 98.5(0.8) | 3.02(1.60 to 4.43) | 0.02(-1.98 to 2.02) | 0.98 |
| **Education level of the familial participant** | |  |  |  |  |  |  |  |  |  |  |
|  | ≤Primary school | 50 | 96.3(1.3) | 97.0(1.6) | 0.67(-2.07 to 3.41) | 39 | 94.4(1.6) | 96.8(1.7) | 2.72(-0.41 to 5.86) | -2.43(-6.70 to 1.85) | 0.26 |
|  | Secondary education | 117 | 94.5(0.8) | 97.1(0.9) | 2.47(0.81 to 4.13) | 106 | 95.3(1.0) | 97.8(1.0) | 2.69(0.95 to 4.44) | -0.14(-2.59 to 2.30) | 0.91 |
|  | High school | 51 | 93.2(1.5) | 97.5(1.2) | 4.30(1.71 to 6.89) | 57 | 94.9(1.1) | 100.7(1.1) | 5.60(3.14 to 8.07) | -0.73(-4.32 to 2.86) | 0.69 |
|  | College or above | 44 | 94.2(1.3) | 97.5(1.2) | 3.49(0.47 to 6.50) | 60 | 94.0(1.2) | 96.4(1.3) | 2.32(-0.27 to 4.90) | 0.39(-3.60 to 4.39) | 0.85 |
| **Adults** | |  |  |  |  |  |  |  |  |  |  |
| **Age group** | |  |  |  |  |  |  |  |  |  |  |
|  | <40 | 171 | 108.3(1.1) | 110.0(1.0) | 1.92(0.34 to 3.50) | 167 | 106.7(1.0) | 109.6(1.1) | 3.21(1.63 to 4.80) | -1.10(-3.33 to 1.13) | 0.33 |
|  | ≥40 | 91 | 121.5(1.8) | 118.3(2.0) | -2.41( -5.27 to 0.44) | 95 | 120.1(2.1) | 121.3(2.2) | 2.30(-0.48 to 5.07) | -4.20(-8.16 to -0.25) | 0.04 |
| **Gender** | |  |  |  |  |  |  |  |  |  |  |
|  | Male | 97 | 118.9(1.7) | 119.8(1.5) | 1.50(-0.95 to 3.95) | 97 | 119.4(1.7) | 122.3(1.8) | 3.52(1.10 to 5.94) | -1.70(-5.02 to 1.62) | 0.31 |
|  | Female | 165 | 109.4(1.2) | 108.8(1.2) | -0.21(-2.00 to 1.57) | 165 | 106.9(1.3) | 108.8(1.3) | 2.49(0.72 to 4.27) | -2.16(-4.65 to 0.33) | 0.09 |
| **Area** | |  |  |  |  |  |  |  |  |  |  |
|  | County | 78 | 112.7(1.7) | 111.2(1.8) | -1.52(3.94 to 0.90) | 78 | 111.4(2.0) | 112.3(2.2) | 1.53(-0.85 to 3.91) | -4.21(-7.59 to -0.82) | 0.02 |
|  | District | 184 | 113.0(1.3) | 113.6(1.2) | 1.22(-0.55 to 3.00) | 184 | 111.6(1.3) | 114.4(1.3) | 3.48(1.72 to 5.24) | -1.45(-3.96 to 1.07) | 0.26 |
| **Education level** | |  |  |  |  |  |  |  |  |  |  |
|  | ≤Primary school | 50 | 118.7(2.5) | 117.8(2.5) | -0.57(-4.56 to 3.42) | 39 | 120.3(3.4) | 119.4(3.5) | -0.26(-4.92 to 4.39) | -0.14(-6.32 to 6.03) | 0.96 |
|  | Secondary education | 117 | 113.1(1.5) | 113.4(1.4) | 0.52(-1.64 to 2.67) | 106 | 111.7(1.6) | 114.9(1.6) | 3.83(1.63 to 6.04) | -3.39(-6.46 to -0.31) | 0.03 |
|  | High school | 51 | 108.5(2.0) | 108.1(2.0) | 0.84(-2.19 to 3.88) | 57 | 109.2(1.8) | 111.8(1.9) | 3.56(0.71 to 6.41) | -2.75(-6.96 to 1.46) | 0.20 |
|  | College or above | 44 | 110.8(2.6) | 110.9(2.2) | 0.80(2.36 to 3.95) | 60 | 107.7(2.3) | 110.0(2.7) | 2.43(-0.22 to 5.07) | -0.82(-5.08 to 3.43) | 0.70 |
| **Blood pressure status** | |  |  |  |  |  |  |  |  |  |  |
|  | Normotensive | 212 | 107.4(0.8) | 108.7(0.8) | 1.17(-0.19 to 2.53) | 221 | 107.0(0.8) | 109.1(0.8) | 2.62(1.28 to 3.95) | -1.36(-3.25 to 0.53) | 0.16 |
|  | Hypertensive | 50 | 136.1(2.3) | 132.0(2.4) | -2.86(-8.03 to 2.31) | 40 | 135.9(3.3) | 138.8(3.3) | 3.60(-1.89 to 9.09) | -5.80(-13.28 to 1.69) | 0.13 |
| **BMI^a^** | |  |  |  |  |  |  |  |  |  |  |
|  | <24 | 136 | 107.6(1.2) | 108.8(1.3) | 1.72(-0.17 to 3.61) | 156 | 107.5(1.2) | 109.2(1.3) | 2.32(0.58 to 4.06) | -0.42(-3.00 to 2.15) | 0.75 |
|  | ≥24 | 126 | 118.6(1.6) | 117.0(1.4) | -0.98(-3.19 to 1.22) | 106 | 117.4(1.8) | 120.3(1.8) | 3.60(1.22 to 5.99) | -5.01(-8.28 to -1.74) | 0.003 |
| **Smoking** | |  |  |  |  |  |  |  |  |  |  |
|  | No | 206 | 111.3(1.1) | 110.7(1.0) | -0.14(-1.72 to 1.44) | 213 | 110.4(1.2) | 112.8(1.3) | 2.84(1.30 to 4.39) | -2.51(-4.69 to -0.32) | 0.02 |
|  | Yes | 56 | 118.8(2.3) | 121.0(2.2) | 2.64(-0.87 to 6.15) | 49 | 116.4(2.0) | 118.1(2.1) | 2.99(-0.68 to 6.66) | -0.87(-5.88 to 4.15) | 0.73 |
| **Relationship** | |  |  |  |  |  |  |  |  |  |  |
|  | Parents | 207 | 110.9(1.1) | 111.4(1.0) | 0.99(-0.59 to 2.57) | 217 | 108.7(1.1) | 111.1(1.1) | 2.70(1.17 to 4.22) | -1.52(3.69 to 0.66) | 0.17 |
|  | Grandparents | 50 | 121.3(2.2) | 119.1(2.5) | -1.16(-4.76 to 2.44) | 43 | 125.2(2.9) | 127.5(3.0) | 3.85(-0.13 to 7.82) | -4.53(-9.97 to 0.91) | 0.10 |
|  | Other^c^ | 5 |  |  | - | 2 |  |  | - | - | - |

^a^P<0.05 for the interaction with time and group.

^b^N denotes the number of participants included in the analysis.

^c^Not being analyzed for too small sample size.

^d^Comparison of the means between baseline and 12-month follow-up. Positive values=increases from baseline to 12-month follow-up; negative values=reductions from baseline to 12-month follow-up. Results were obtained from mixed linear model taking into account of the hierarchical structure of data.

^e^Comparison between intervention and control groups in the changes from baseline to 12-month follow-up. Positive values=the intervention group had a greater increase or less decrease from baseline to 12-month follow-up than the control group; negative values=the intervention group has a greater decrease or smaller increase from baseline to 12-month follow-up than the control group. Results were adjusted for age, gender, body mass index (body weight in children instead), district or county, physical exercise, and education level (the education level of the familial participant in children instead). In adults, additional adjustments were made for smoking, alcohol consumption, and relationship with the child. Blood pressure values were further adjusted for outdoor temperature.

Table S6. Diastolic blood pressure (mm Hg) by subgroup.^a-e^

|  | | Intervention group | | | | Control group | | | | Adjusted difference in change^e^ | |
| --- | --- | --- | --- | --- | --- | --- | --- | --- | --- | --- | --- |
|  | | N^b^ | Baseline mean(SE) | 12 months mean(SE) | Change^d^(95%CI) | N^b^ | Baseline  mean(SE) | 12 months  mean(SE) | Change^d^(95%CI) | Intervention VS Control (95%CI) | *P* |
| **Children** | |  |  |  |  |  |  |  |  |  |  |
| **Gender** | |  |  |  |  |  |  |  |  |  |  |
|  | Boys | 142 | 60.4(0.6) | 60.7(0.5) | 0.31(-1.06 to 1.67) | 146 | 58.7(0.6) | 60.3(0.6) | 1.51(0.16 to 2.86) | -1.31(-3.28 to 0.65) | 0.19 |
|  | Girls | 120 | 60.6(0.6) | 60.5(0.6) | -0.17(-1.64 to 1.30) | 116 | 59.1(0.8) | 60.5(0.7) | 1.38(-0.11 to 2.87) | -1.43(-3.54 to 0.69) | 0.18 |
| **Area** | |  |  |  |  |  |  |  |  |  |  |
|  | County | 78 | 59.9(0.8) | 60.5(0.8) | 0.63(-1.03 to 2.29) | 78 | 58.1(0.8) | 61.4(0.7) | 3.40(1.74 to 5.06) | -2.88(-5.31 to -0.46) | 0.02 |
|  | District | 184 | 60.8(0.5) | 60.7(0.4) | -0.13(-1.36to 1.09) | 184 | 59.2(0.6) | 59.9(0.6) | 0.61(-0.62 to 1.84) | -0.68(-2.44 to 1.08) | 0.45 |
| **Education level of the familial participant** | |  |  |  |  |  |  |  |  |  |  |
|  | ≤Primary school | 50 | 62.1(0.9) | 59.9(1.0) | -2.25(-4.52 to 0.02) | 39 | 58.8(1.2) | 59.5(1.5) | 0.84(-1.76 to 3.43) | -2.72(-6.34 to 0.93) | 0.14 |
|  | Secondary education | 117 | 60.1(0.7) | 60.8(0.6) | 0.65(-0.80 to 2.11) | 106 | 59.2(0.7) | 60.8(0.7) | 1.56(0.04 to 3.09) | -0.92(-3.05 to 1.22) | 0.40 |
|  | High school | 51 | 59.8(1.0) | 61.3(0.7) | 1.48(-0.82 to 3.79) | 57 | 58.8(1.1) | 61.7(0.9) | 2.81(0.62 to 5.01) | -1.34(-4.59 to 1.91) | 0.42 |
|  | College or above | 44 | 60.7(0.8) | 60.3(1.0) | -0.25(-2.82 to 2.31) | 60 | 58.5(1.1) | 58.9(0.9) | 0.36(-1.84 to 2.55) | -1.03(-4.43 to 2.37) | 0.55 |
| **Adults** | |  |  |  |  |  |  |  |  |  |  |
| **Age group** | |  |  |  |  |  |  |  |  |  |  |
|  | <40 | 171 | 71.0(0.8) | 71.8(0.8) | 0.62(-0.71 to 1.96) | 167 | 68.9(0.7) | 71.5(0.8) | 2.70(1.36 to 4.04) | -1.76(-3.58 to 0.06) | 0.06 |
|  | ≥40 | 91 | 76.6(1.2) | 73.6(1.2) | -2.45(-4.35 to -0.56) | 95 | 76.1(1.3) | 76.6(1.4) | 0.91(-0.93 to 2.76) | -2.99(-5.69 to -0.30) | 0.03 |
| **Gender** | |  |  |  |  |  |  |  |  |  |  |
|  | Male | 97 | 76.9(1.2) | 76.2(1.0) | -0.68(-2.52 to 1.16) | 97 | 75.7(1.2) | 77.6(1.3) | 2.35(0.52 to 4.17) | -3.09(-5.65 to-0.52) | 0.02 |
|  | Female | 165 | 70.7(0.8) | 70.2(0.8) | -0.34(-1.72 to 1.03) | 165 | 69.1(0.7) | 70.8(0.8) | 1.87(0.51 to 3.24) | -1.86(-3.77 to 0.04) | 0.06 |
| **Area^a^** | |  |  |  |  |  |  |  |  |  |  |
|  | County | 78 | 73.2(1.2) | 71.8(1.3) | -1.60(-3.25 to 0.04) | 78 | 70.7(1.1) | 72.5(1.3) | 1.85(0.23 to 3.46) | -4.97(-7.19 to -2.74) | <0.001 |
|  | District | 184 | 72.9(0.9) | 72.7(0.8) | -0.01(-1.42 to 1.39) | 184 | 71.9(0.8) | 73.7(0.9) | 2.13(0.73 to 3.52) | -1.10(-3.08 to 0.89) | 0.28 |
| **Education level** | |  |  |  |  |  |  |  |  |  |  |
|  | ≤Primary school | 50 | 75.4(1.5) | 74.6(1.5) | -0.34(-3.24 to 2.56) | 39 | 74.3(2.1) | 73.9(2.1) | -0.13(-3.51 to 3.25) | -0.46(-4.91 to 4.00) | 0.84 |
|  | Secondary education | 117 | 72.7(1.1) | 73.4(1.0) | 0.48(-1.19 to 2.16) | 106 | 71.4(1.0) | 74.4(1.1) | 3.11(1.40 to 4.82) | -2.71(-5.10 to-0.33) | 0.03 |
|  | High school | 51 | 70.8(1.5) | 67.8(1.4) | -2.56(-5.00 to -0.12) | 57 | 70.8(1.2) | 72.3(1.4) | 1.89(-0.40 to 4.19) | -4.04(-7.37 to -0.72) | 0.02 |
|  | College or above | 44 | 73.4(1.7) | 72.6(1.3) | -0.95(-3.26 to 1.35) | 60 | 70.6(1.5) | 72.0(1.6) | 1.56(-0.38 to 3.50) | -2.35(-5.48 to 0.77) | 0.14 |
| **Blood pressure status** | |  |  |  |  |  |  |  |  |  |  |
|  | Normotensive | 212 | 69.7(0.6) | 70.0(0.6) | 0.14(-0.94 to 1.23) | 221 | 68.8(0.5) | 70.4(0.6) | 1.91(0.85 to 2.97) | -1.64(-3.12 to -0.15) | 0.03 |
|  | Hypertensive | 50 | 86.9(1.6) | 83.6(1.6) | -2.99(-6.62 to 0.64) | 40 | 86.4(1.8) | 88.8(1.8) | 2.49(-1.38 to 6.36) | -5.12(-10.30 to 0.06) | 0.05 |
| **BMI** | |  |  |  |  |  |  |  |  |  |  |
|  | <24 | 136 | 69.7(0.8) | 69.4(0.8) | -0.14(-1.58 to 1.31) | 156 | 68.9(0.8) | 70.5(0.9) | 1.85(0.52 to 3.18) | -1.61(-3.51 to 0.30) | 0.10 |
|  | ≥24 | 126 | 76.6(1.1) | 75.5(1.0) | -0.88(-2.57 to 0.81) | 106 | 75.4(1.1) | 77.3(1.1) | 2.26(0.42 to 4.09) | -3.51(-6.00 to -1.03) | 0.006 |
| **Smoking** | |  |  |  |  |  |  |  |  |  |  |
|  | No | 206 | 72.0(0.8) | 71.2(0.7) | -0.54(-1.76 to 0.69) | 213 | 71.0(0.7) | 72.6(0.8) | 1.66(0.47 to 2.86) | -1.94(-3.62 to -0.27) | 0.02 |
|  | Yes | 56 | 76.7(1.6) | 77.0(1.4) | -0.17(-2.71 to 2.37) | 49 | 73.8(1.6) | 76.7(1.7) | 3.73(1.07 to 6.39) | -4.71(-8.37 to -1.06) | 0.01 |
| **Relationship** | |  |  |  |  |  |  |  |  |  |  |
|  | Parents | 207 | 72.5(0.8) | 72.4(0.7) | 0.00(-1.24 to 1.24) | 217 | 70.4(0.7) | 72.6(0.8) | 2.30(1.11 to 3.49) | -2.00(-3.68 to -0.32) | 0.02 |
|  | Grandparents | 50 | 75.1(1.4) | 72.8(1.5) | -1.96(-4.43 to 0.51) | 43 | 76.8(1.7) | 77.1(2.0) | 0.95(-1.79 to 3.68) | -2.96(-6.79 to 0.86) | 0.13 |
|  | Other^c^ | 5 | - | - | - | 2 | - | - | - | - | - |

^a^P<0.05 for the interaction with time and group.

^b^N denotes the number of participants included in the analysis.

^c^Not being analyzed for too small sample size.

^d^Comparison of the means between baseline and 12-month follow-up. Positive values=increases from baseline to 12-month follow-up; negative values=reductions from baseline to 12-month follow-up. Results were obtained from mixed linear model taking into account of the hierarchical structure of data.

^e^Comparison between intervention and control groups in the changes from baseline to 12-month follow-up. Positive values=the intervention group had a greater increase or less decrease from baseline to 12-month follow-up than the control group; negative values=the intervention group has a greater decrease or smaller increase from baseline to 12-month follow-up than the control group. Results were adjusted for age, gender, body mass index (body weight in children instead), district or county, physical exercise, and education level (the education level of the familial participant in children instead). In adults, additional adjustments were made for smoking, alcohol consumption, and relationship with the child. Blood pressure values were further adjusted for outdoor temperature.
